# Changes in patient visits and diagnoses in a large academic center during the COVID-19 pandemic

**DOI:** 10.1186/s12886-021-01886-7

**Published:** 2021-03-20

**Authors:** Meghan K. Berkenstock, Paulina Liberman, Peter J. McDonnell, Benjamin C. Chaon

**Affiliations:** 1 Wilmer Eye Institute, Johns Hopkins School of Medicine, 600 N. Wolfe Street, Maumenee Building, Third Floor, Baltimore, MD 21287 USA; 2 Departamento de Oftalmología, Escuela de Medicina, Pontificia Universidad Católica de Chile, Santiago, Chile

**Keywords:** COVID-19, Epiphenomena, Ocular diagnoses, Ophthalmology, Telemedicine

## Abstract

**Background:**

To minimize the risk of viral transmission, ophthalmology practices limited face-to-face encounters to only patients with urgent and emergent ophthalmic conditions in the weeks after the start of the COVID-19 epidemic in the United States. The impact of this is unknown.

**Methods:**

We did a retrospective analysis of the change in the frequency of ICD-10 code use and patient volumes in the 6 weeks before and after the changes in clinical practice associated with COVID-19.

**Results:**

The total number of encounters decreased four-fold after the implementation of clinic changes associated with COVID-19. The low vision, pediatric ophthalmology, general ophthalmology, and cornea divisions had the largest total decrease of in-person visits. Conversely, the number of telemedicine visits increased sixty-fold. The number of diagnostic codes associated with ocular malignancies, most ocular inflammatory disorders, and retinal conditions requiring intravitreal injections increased. ICD-10 codes associated with ocular screening exams for systemic disorders decreased during the weeks post COVID-19.

**Conclusion:**

Ophthalmology practices need to be prepared to experience changes in practice patterns, implementation of telemedicine, and decreased patient volumes during a pandemic. Knowing the changes specific to each subspecialty clinic is vital to redistribute available resources correctly.

**Supplementary Information:**

The online version contains supplementary material available at 10.1186/s12886-021-01886-7.

## Background

In the wake of the spread of COVID-19 in the United States, the practice of ophthalmology has undergone a dramatic and unprecedented shift. The inherent risk of transmission is high due to the need for patient proximity during an ophthalmic exam or surgery, which has led to at least one outbreak in Norway [1–13]. In March 2020, the American Academy of Ophthalmology (AAO) issued practice guidelines and released a list of emergent ophthalmic surgical procedures to be continued during the COVID-19 pandemic [14, 15]. Subsequently, the rescheduling of routine ocular examinations and limiting procedures to those deemed urgent or emergent has been the basis of many recent publications [16–27]. Each has offered specialty specific guidelines to limit transmission moving forward, while allowing for the gradual increase in ophthalmic exams, intraocular injections, and surgeries during the COVID-19 pandemic [16–27].

Adherence to these recommendations has resulted in an estimated 75% or more reduction of in-person clinic volumes [28]. Concurrently, the implementation of telemedicine visits resulted in an alternative way to provide continued care [3, 25, 29–31]. However, the exact reduction of in-patient visits, increase in telemedicine encounters, and the change in the types of diagnoses seen within in an ophthalmology clinic before and after COVID-19 remains unknown. Herein, we describe these changes in clinic volume over all subspecialists in an academic eye clinic as epiphenomena of COVID-19 in ophthalmic practice.

## Methods

**Fig. 1:**
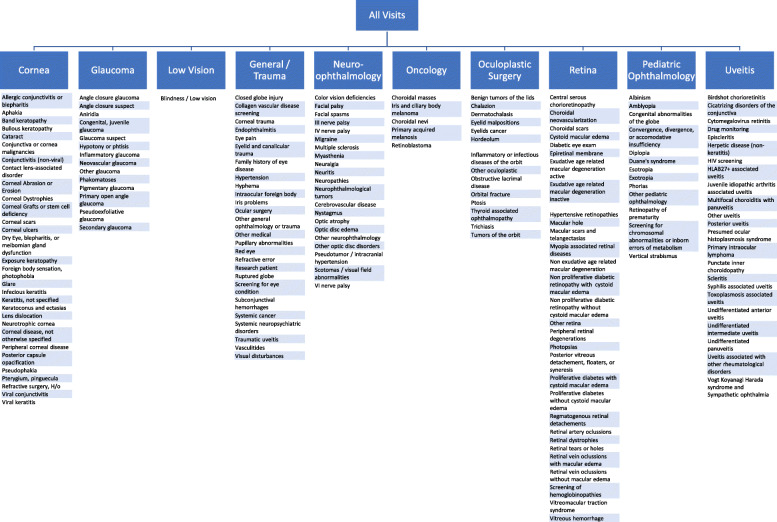
Diagnostic Categories by Subspecialty

This was a retrospective analysis of all the ICD-10 diagnosis codes listed in patient encounters from all of the divisions within the Wilmer Eye Institute, main site and satellite clinics, during the 6 weeks prior to (2/17/20 through 3/21/20) and the 6 weeks after the start of emergency visits (3/22/20 through 4/30/20) in response to COVID-19. Immediate institution of a policy providing care for emergent and urgent ophthalmic care was inititated by department leadership in conjuction with Johns Hopkins Health System administration. There was no progressive ramp down of clinical activities. Patients were rescheduled for 4 weeks after their previously booked clinic appointment during this period. If there were any questions on the severity or condition being treated, the treating physician was contacted to advise on the timing of follow-up. ICD-10 codes allow for specification of eye laterality, affected site, and other complications in systemic diseases. All ICD-10 codes for the same diagnosis were consolidated and divided into categories based on ophthalmic subspecialty service at the Wilmer Eye Institute: cornea, oculoplastic surgery, retina, uveitis, pediatric ophthalmology, neuro-ophthalmology, glaucoma, trauma and general ophthalmology, low vision, and oncology. Included with general ophthalmology were ICD-10 codes associated with screening for systemic diseases affecting the eye. The ICD-10 codes were then subdivided into 177 categories based on anatomic location or mechanism of disease (Supplement 1 and Fig. 1). To compare frequency of visits by subspecialty, retina and oncology were merged due to the dual training of our ocular oncologists. Since they also see other retinal conditions in their clinical practice, it was impossible to completely isolate ocular oncology and retina department visits. The rest of the subspecialties were divided in the same way as previously described.

In addition, the number of patient encounters overall and per department were recorded and subdivided into in-person and telemedicine types. Diagnostic categories and encounter frequencies were analyzed; comparisons were evaluated using the χ2 test or Fisher exact test if expected counts within a category were < 5. Statistical analyses were performed using SPSS software version 22.0 (IBM, Armonk, NY, USA). We considered a *p*-value below 0.05 to be statistically significant. This study was reviewed and approved by the Johns Hopkins Hospital Institutional Review Board (IRB00250067).

## Results

In the 6 weeks prior to the start of emergency visits, there were 25,336 completed in-person visits, which resulted in the use of 5261 ICD-10 codes. This is compared with the drastic reduction of patients seen in the 6 weeks thereafter when 5672 in-person visits were completed, which included 2207 ICD-10 codes.

**Fig. 2:**
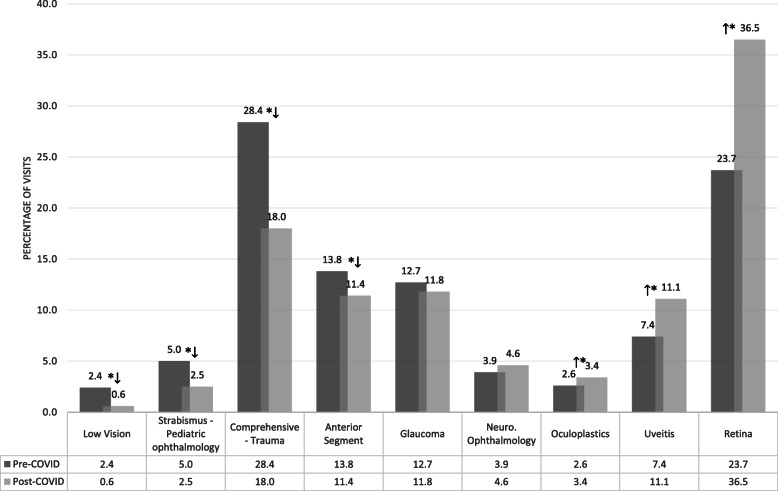
A bar graph depicting the change in the percentage of in-person visits by subspecialty in each study period. Significant differences are marked with an asterisk (*)

Analyzing the change in the proportional frequency between the 6 weeks after the start of emergency visits and the 6 weeks prior, the retina, uveitis, and oculoplastic surgery divisions showed a significant increase in in-person visits (*p* < 0.001, < 0.001, and 0.032, respectively). Low vision, pediatric ophthalmology, general ophthalmology, and the cornea division percentage of in-person visits significantly decreased (*p* < 0.001, < 0.001, < 0.001, 0.001 respectively). There was no significant change in appointments within the glaucoma or neuro-ophthalmology divisions (Fig. 2). In contrast, the amount of telemedicine visits increased 60 fold in the 6 weeks after the start of emergency visits and accounted for 7.6% of total visits during that period (*p* < 0.001).

**Fig. 3:**
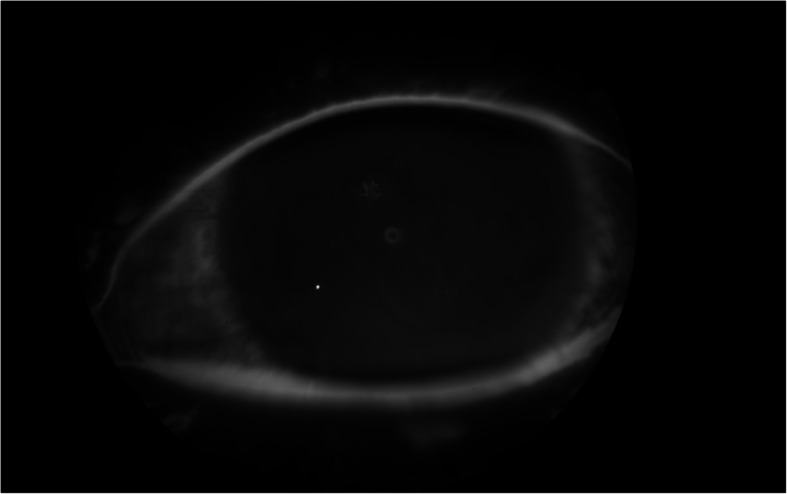
External photograph of pseudodendrites as seen in a patient with herpetic keratitis

We found significant differences in the proportion of ICD-10 codes used in between the pre- and the post-COVID-19 change to emergency in-patient visits (Supplement 2 and Table 1). Of the anterior segment disorders, conjunctival and corneal malignancies, complications of contact lens use, peripheral corneal disease, viral keratitis (Fig. 3) and corneal ulcer ICD-10 codes were used significantly more frequently in the 6 weeks after the shift to emergency visits. Conversely, pterygium and pinguecula, cataract, and refractive surgery ICD-10 codes were used significantly less during the same time period. Specifically related to glaucomatous conditions, there was a significant increase in the use of neovascular, juvenile or congenital, uveitic, and secondary glaucoma ICD-10 codes in the weeks after COVID-19 with a concurrent decline of glaucoma suspect and angle-closure suspect codes.

**Table 1:** The most significant changes in diagnostic codes by ophthalmic subspecialty

ANTERIOR SEGMENT						
**TREND**	**DISEASE CATEGORY**	**PRE-COVID n** ***%***		**POST-COVID n** ***%***		***p*** **value**
↑	**Conjunctival or Corneal Malignancy**	7	*0.0*	10	*0.2*	0.000
	**Contact Lens-Associated Disorder**	7	*0.0*	8	*0.2*	0.002
	**Peripheral Cornea Disease**	19	*0.1*	15	*0.3*	0.000
	**Corneal Ulcer**	186	*0.7*	134	*2.5*	0.000
↓	**Pterigium and Pinguecula**	123	*0.5*	9	*0.2*	0.002
	**Cataract**	3851	*15.2*	240	*4.4*	0.000
	**Refractive surgery**	154	*0.6*	8	*0.2*	0.000
**GLAUCOMA**						
**TREND**	**DISEASE CATEGORY**	**PRE-COVID n** ***%***		**POST-COVID n** ***%***		***p*** **value**
↑	**Neovascular Glaucoma**	73	*0.3*	51	*1.0*	0.000
	**Congenital and Juvenile Glaucoma**	38	*0.2*	22	*0.4*	0.000
	**Inflammatory/ Uveitic Glaucoma**	88	*0.3*	51	*1.0*	0.000
	**Secondary Glaucomas**	154	*0.6*	71	*1.3*	0.000
↓	**Angle Closure Suspects**	194	*0.8*	27	*0.5*	0.048
	**Glaucoma Suspects**	2654	*10.5*	350	*6.3*	0.000
**LOW VISION**						
**TREND**	**DISEASE CATEGORY**	**PRE-COVID n** ***%***		**POST-COVID n** ***%***		***p*** **value**
↓	**Legal Blindness and Low Vision**	166	*0.7*	6	*0.1*	0.000
**GENERAL OPHTHALMOLOGY / TRAUMA**						
**TREND**	**DISEASE CATEGORY**	**PRE-COVID n** ***%***		**POST-COVID n** ***%***		***p*** **value**
↑	**Ruptured Globe**	43	*0.2*	40	*0.8*	0.000
	**Intraocular Foreign Body**	16	*0.1*	10	*0.2*	0.008
	**Vasculitides, ocular exam to rule out complications**	15	*0.1*	9	*0.2*	0.014
	**Research Patient Encounters**	21	*0.1*	10	*0.2*	0.025
	**Corneal Trauma**	74	*0.3*	34	*0.6*	0.000
	**Endophthalmitis**	38	*0.2*	17	*0.3*	0.007
↓	**Screening exam for an eye condition**	169	*0.7*	20	*0.4*	0.016
	**Refractive Error**	3282	*13.0*	89	*1.7*	0.000
	**Family History of Eye Disease, Screening**	35	*0.1*	0	*0.0*	0.007
**NEURO-OPHTHALMOLOGY**						
**TREND**	**DISEASE CATEGORY**	**PRE-COVID n** ***%***		**POST-COVID n** ***%***		***p*** **value**
↑	**Optic Disc Edema**	74	*0.3*	41	*0.8*	0.000
	**Sixth Nerve Palsy**	28	*0.1*	14	*0.3*	0.006
	**Optic Neuritis**	22	*0.1*	11	*0.2*	0.014
	**Intracranial Hypertension**	62	*0.2*	30	*0.6*	0.000
↓	**Nystagmus**	77	*0.3*	5	*0.1*	0.008
**OCULAR ONCOLOGY**						
**TREND**	**DISEASE CATEGORY**	**PRE-COVID n** ***%***		**POST-COVID n** ***%***		***p*** **value**
↑	**Choroidal Mass**	88	*0.3*	32	*0.6*	0.006
↓	**Choridal Nevus**	187	*0.7*	18	*0.3*	0.001
**OCULOPLASTIC SURGERY**						
**TREND**	**DISEASE CATEGORY**	**PRE-COVID n** ***%***		**POST-COVID n** ***%***		***p*** **value**
↓	**Nasolacrimal Duct Obstruction**	175	*0.7*	19	*0.4*	0.006
	**Benign Eyelid Lesions**	133	*0.5*	13	*0.2*	0.008
	**Eyelid Malpositions**	118	*0.5*	11	*0.2*	0.009
	**Dermatochalasis**	110	*0.4*	2	*0.0*	0.000
**RETINA**						
**TREND**	**DISEASE CATEGORY**	**PRE-COVID n** ***%***		**POST-COVID n** ***%***		***p*** **value**
↑	**Venous Oclussions with Macular Edema**	373	*1.5*	280	*5.1*	0.000
	**Active, Exudative Age-Related Macular Degeneration**	1099	*4.3*	778	*12.9*	0.000
	**Choroidal neovascularization**	156	*0.6*	100	*1.9*	0.000
	**Proliferative Diabetic Retinopathy with Cystoid Macular Edema**	360	*1.4*	212	*3.9*	0.000
↓	**Choroidal Scars**	148	*0.6*	13	*0.2*	0.002
	**Retinal Dystrophies**	252	*1.0*	18	*0.3*	0.000
	**Diabetic Eye Exams**	1145	*4.5*	59	*1.1*	0.000
	**Ocular Exam in Patients with Hemoglobinopathies**	22	*0.1*	0	*0.0*	0.023
**PEDIATRIC OPHTHALMOLOGY**						
**TREND**	**DISEASE CATEGORY**	**PRE-COVID n** ***%***		**POST-COVID n** ***%***		***p*** **value**
↓	**Esotropia**	321	*1.3*	13	*0.2*	0.000
	**Exotropia**	315	*1.2*	9	*0.2*	0.000
	**Phorias**	51	*0.2*	1	*0.0*	0.004
	**Ocular Exam in Patients with Chromosomal Abnormalities or Inborn Errors of Metabolism**	26	*0.1*	0	*0.0*	0.016
**UVEITIS**						
**TREND**	**DISEASE CATEGORY**	**PRE-COVID n** ***%***		**POST-COVID n** ***%***		***p*** **value**
↑	**Primary Intraocular Lymphoma**	7	*0.0*	7	*0.1*	0.005
	**Vogt-Koyanagi-Harada Syndrome, Sympathetic Ophthalmia**	12	*0.0*	10	*0.2*	0.000
	**Traumatic Iritis**	19	*0.1*	14	*0.3*	0.000
	**Undifferentiated Intermediate Uveitis**	51	*0.2*	30	*0.6*	0.000
↓	**Ocular Exam in Patients with HIV**	56	*0.2*	4	*0.1*	0.031

In the neuro-ophthalmology division, optic disc edema, sixth nerve palsies, optic neuritis, and pseudotumor ICD-10 codes were used more frequently post-COVID-19; whereas, the code for nystagmus decreased significantly in frequency. Within the retina division, there was a significant increase in ICD-10 codes associated with the need for injections, such as vein occlusions with macular edema, active wet age-related macular degeneration, active choroidal neovascular membranes (CNVM), and proliferative diabetes with cystoid macular edema (CME). Conversely, ICD-10 codes associated with chronic chorioretinal diseases and retinal screening exams significantly decreased, which included choroidal scars, retinal dystrophies, diabetic eye exams, and screening for hemoglobinopathies.

The uveitis division showed a decrease in ICD-10 codes associated with screening for ocular complications of HIV disease, but no other uveitis associated codes declined. Rather, ICD-10 codes for primary intraocular lymphoma, Vogt-Koyanagi-Harada disease and sympathetic ophthalmia, traumatic uveitis, non-corneal herpetic diseases, and intermediate uveitis were used signficantly more than in the pre-COVID-19 time period. Ocular oncology, as well, showed a significant decrease in the use of choroidal nevus screening codes and showed a signficant increase in the use of choroidal mass codes.

None of the codes associated with pediatric ophthalmology, low vision, and oculoplastics had a proportional increase after the change in in-person visits associatd with COVID-19 were implemented. The ICD-10 codes that declined the most were ocular screenings for chromosomal abnormalities, phorias, and strabismus. In oculoplastic surgery, ICD-10 codes for dermatochalasis, eyelid malpositions, benign tumors of the lids, and obstructive lacrimal duct disease also declined. Finally, ICD-10 codes associated with low vision decreased 27 fold in the 6 weeks post-COVID-19.

Ocular trauma showed a proportional increase in ruptured globes and intraocular foreign bodies in the 6 weeks after the COVID-19 changes. ICD-10 codes for screening for eye or systemic medical disorders, a family history of eye disease, and refractive error were used significantly less frequently than in the 6 weeks pre-COVID-19.

## Discussion

The recommendations by the AAO to limit routine ophthalmic examinations and restrict in-patient evaluations to urgent and emergent ocular conditions have been implemented in all of our divisions during COVID 19 as evidenced by the 60 fold increase in telemedicine visits in the weeks after COVID-19 [14, 15]. While the implementation of telehealth visits was being piloted prior to the epidemic, the need to limit in-person evaluations led to rapid wide-spread adoption in most of the divisions. Equally striking, was the significant decline in ophthalmic screening exams for systemic disorders, research study visits, and low vision services. As a whole, ophthalmic conditions leading to immediate vision change, pain and photophobia, ocular malignancies, and trauma continued to be seen in-person.

The specific ICD-10 code frequency changes within each subspecialty requires individual discussion. For conditions affecting the cornea, a significant increase in the percentage of patients seen with ulcers, complications of contact lens use, and peripheral diseases including ulcerative keratitis occurred in the 6 weeks following the start of emergent visits. This again highlights the continued presentation of patients with ocular pain, photophobia, and change in vision. Despite reports of conjunctivitis and red eyes as symptoms of COVID-19, all types of conjunctivitis continued to have a statistically significant decrease in the 6 weeks following COVID-19, except viral conjunctivitis, which decreased in frequency but not significantly [24–32]. The largest statistically significant decreases were seen in post-cataract follow-up visits, as evidenced by a decrease in ICD-10 codes for posterior capsular opacification and pseudophakia. All of the pre-operative and non-urgent follow-up rescheduling led to a 78% drop in visits.

Conversely, as noted by Skalet et al., the time-sensitive nature for evaluation and continued treatment of ocular malignancies requires appointment flexibility and patient-centered judgement on the risk of delaying care during COVID-19 with possible tumor progression [16]. In our department the percentage of choroidal masses and corneal malignancies diagnoses increased, whereas screening exams for choroidal nevi decreased significantly. Oculoplastic surgery evaluations were also dramatically decreased with the rescheduling of elective visits given the corresponding drop in use of dermatochalasis, benign eyelid lesions, and ptosis ICD-10 codes. While the frequency of stye ICD-10 codes increased, chalazions decreased significantly with postponement of non-urgent surgeries.

Neuro-ophthalmic conditions that increased significantly in the weeks following COVID-19 caused diplopia, headache, and loss of vision. On the other hand, exams for nystagmus decreased significantly, reflecting patient presentation to assess acute changes in vision.

Similarly, retinal conditions resulting in vision loss continued to be evaluated at statistically increased frequencies, including macular holes, retinal detachments, vitreous hemorrhages, or cystoid macular edema. Chronic conditions or those requiring long-term monitoring decreased in the weeks post-COVID. Retinal conditions at high risk for complications, such as inactive wet macular degeneration, and conditions requiring continuation of intravitreal injections, such as diabetic macular edema, continued to be seen frequently in alignment with recommendations for continued treatment during COVID-19 [19, 21, 26]. One of the highest risk groups for complications related to COVID-19 infection are those over 65 years-old or with pre-existing health conditions, which describes the demographics of many patients receiving intravitreal injections [19, 26, 33]. Patients and providers needed to weigh the risk of possible COVID-19 exposure with disease progression or the development of additional ocular complications with the postponement of anti-vascular endothelial growth factor (anti-VEGF) injections and possible disease progression [19]. Interestingly, the codes for neovascular glaucoma (NVG) significantly increased in the 6 weeks post-COVID. Thus, a further study is needed to assess the relationship between the increase in NVG ICD-10 codes and the postponement of anti-VEGF injections during the pandemic.

Overall, the majority of ICD-10 codes for glaucomatous conditions increased in frequency significantly post-COVID 19, reflecting the continued need for in-person intraocular pressure monitoring [18]. In comparison, pediatric ophthalmology experienced a decrease in routine screening for ocular disorders in patients with congenital or chromosomal abnormalities and strabismus evaluations, but the retinopathy of prematurity (ROP) screening code frequency did not significantly decrease. Guidelines for seeing pediatric ophthalmology patients have been offered and include patients risk for amblyopia, requiring evaluation for leukocoria or congenital glaucoma, infection, or nystagmus [34]. Although only five ICD-10 codes were used in the 6 weeks after COVID-19 for ROP, it still illustrates the continued need for inpatient ophthalmic consultations during COVID-19.

In term of in-person visits, the number significantly increased for the uveitis division given the reliance on slit lamp evaluation for cell and flare [20]. Correspondingly, there was a significant increase in the numbers of ICD-10 codes for all anatomic locations of undifferentiated uveitis and scleritis. Concurrently, uveitic glaucoma ICD-10 codes significantly increased in the weeks after COVID-19. As with NVG, assessing if a relationship between the increase in ICD-10 code use for ocular inflammatory diseases and uveitic glaucoma would be another area for future research. As in other divisions, screening exams for ocular complications of rheumatologic conditions or HIV were significantly decreased. The frequency of high risk medication use and monitoring was the same in the weeks prior to and after COVID-19, but the decrease in number was not significant. This group included patients on immunosuppressive medications prescribed to treat ocular inflammation, illustrating the need to safely continue these medications, in-person evaluations, and lab draws to in the time of COVID-19 [20, 35]. Interestingly, all types of herpetic eye diseases increased in frequency significantly in the 6 weeks after COVID-19. Herpetic disease has been associated with depression as a trigger for reactivation, which has also been noted to increase during COVID 19 [36, 37].

Multiple strategies have been employed to slow transmission of the virus, including stay at home orders, limiting non-essential businesses and travel. While these actions promote social distancing, ocular trauma is still occurring [38, 39]. Previous papers have described an overall decrease in ocular trauma with a subset increase in ocular trauma related to home activities during COVID-19. Only Pelligrini et al. noted a stable rate of orbital fractures and open globe injuries. While we could not assess where the ocular trauma occurred using ICD-10 codes alone, we did have an increase in all forms of penetrating and blunt ocular trauma during active stay-at-home orders in Baltimore. Furthermore, there was a statistical increase in the frequency of corneal abrasions and trauma, traumatic iritis, intraocular lens dislocations, and blunt non-penetrating trauma in the 6 weeks after COVID-19. While there was a decrease in use of ICD-10 codes associated with eyelid trauma, orbital fractures, subconjunctival hemorrhages, and red eyes, these were not statistically significant. In short, all types of ocular trauma still presented for emergent care during COVID-19. Because our institute is the only state-designated Level I Eye Trauma Center in the state of Maryland and some bordering states, it is possible that the increase in trauma observed in this analysis occurred as the result of other potential caregivers having closed their practices or otherwise not being available to care for these patients.

A limitation of this study is the common practice of carrying diagnoses forward during follow-up visits, which can induce bias. Although this may change the proportion of active diseases being evaluated in each period, there is no evidence that carrying diagnoses forward would be different in two time intervals studied. The timing of follow-up is another limitation, as all patients were rescheduled to 4 weeks later with the start of the shut down period. Providers were able to advise a shorter follow-up interval for patients based on the diagnosis being treated or the severity of disease. Since only ICD-10 codes were assessed, there was no way to control for selection bias for follow-up given this was on an individual basis. Conversely, the strengths of this report are the large number of visits and ICD-10 codes reviewed over all ophthalmic subspecialties.

Our analysis sheds light on which diseases are more commonly treated in a real-life, state of emergency. It provides generalizable evidence for ophthalmology departments and health systems alike to allocate human resources and materials to meet patient needs during a pandemic. In our population, ocular trauma occurred in equal numbers during both study periods. This illustrates the need to continue ophthalmic operating room availability and to maintain an uninterrupted ophthalmology trauma on-call schedule even as providers are redeployed to provide medical care in other departments. Ocular inflammatory disease follow-up and high risk medication monitoring significantly increased during COVID-19, requiring proper disinfection of clinic equipment and use of personal protective equipment by patients and staff to provide care to high risk and immunosuppressed patients [1–12, 20, 35]. Even in the subspecialty clinics where a significant decrease of visits was noted, diagnostic codes were still used for patients with acute changes in vision. Thus, a provider within each subspecialty division should remain available for in-patient consultation on a daily basis.

One question that will need to be addressed is whether the pandemic has resulted in the avoidance or delay in care for some individuals, who may as a result be at substantial risk for worse ultimate outcomes. Our finding that fewer patients were being screened for HIV-associated retinal disease and that fewer “glaucoma suspects” were seen during this period may mean that pathology in at least a subset of these individuals will have progressed before they are able or willing to present to the ophthalmologist. We suspect, but our study does not allow us to quantify, that there will be an ultimately greater burden of disease as a result of postponed screening visits and timely therapeutic interventions.

## Conclusions

An academic ophthalmology department associated with a tertiary referral hospital should be prepared to experience changes in practice patterns, implementation of telemedicine, and decreased patient volumes during a pandemic. Knowing the changes specific to each subspecialty clinic is vital to correctly redistributing available resources. Looking forward, a cost-effectiveness analysis is needed to discern the best way to address these changes while continuing to provide safe patient care and lessen the economic burden of these trying times.

## Supplementary Information

**Additional file 1: Supplement 1.** ICD-10 Code Consolidation by Ophthalmic Subspecialty.

**Additional file 2: Supplement 2.** All changes in diagnostic codes by ophthalmic subspecialty.

## Data Availability

All data is shown in the manuscript.
