# Supplementary material for: Changes in patient visits and diagnoses in a large academic center during the COVID-19 pandemic
Source: BMC Ophthalmol. 2021 Mar 20;21:139. doi: 10.1186/s12886-021-01886-7 (PMC7980730; doi:10.1186/s12886-021-01886-7)
Supplement: Supplementary file 1 — Additional file 1: Supplement 1. ICD-10 Code Consolidation by Ophthalmic Subspecialty. [file 12886_2021_1886_MOESM1_ESM.docx]

| **Ocular Subspecialty** | **Disease Categories** | **Diagnoses Within the Category** |
| --- | --- | --- |
| **Cornea** | Allergic Conjunctivitis or Blepharitis | - Allergic contact dermatitis - Allergic conjunctivitis (Acute & Chronic) - Vernal - Atopic - Epidemic Keratoconjunctivitis (EKC) - Eczematous dermatitis |
|  | Aphakia | -Aphakia |
|  | Band Keratopathy | - Band-shaped keratopathy - Band keratopathy |
|  | Bullous Keratopathy | - Corneal edema (idiopathic, secondary) - Corneal guttata |
|  | Cataract | - Unspecified - Age-related (nuclear, cortical, combined-forms, posterior-subcapsular, posterior-subcapsular polar) - Cortical - Nuclear sclerotic - Cataract in inflammatory disorder - Incipient - Juvenile nuclear - Localized traumatic - Uveitic |
|  | Conjunctival or Corneal Malignancies | - Squamous cell carcinoma of conjunctiva - Corneal intraepithelial neoplasia - Conjunctival intraepithelial neoplasia - Conjunctival tumor - Carcinoma in situ, conjunctiva - Malignant melanoma of conjunctiva |
|  | Conjunctivitis, Non-viral | - Unspecified conjunctivitis (acute & chronic) - Follicular (acute & chronic) - Purulent - Bacterial - Pseudomembranous - Papillary |
|  | Contact Lens-Associated Disorder | - Sterile keratitis associated with contact lens - Contact lens induced keratopathy - Contact lens overwear |
|  | Corneal Abrasion or Erosion | - Corneal abrasion (initial, subsequent, sequelae, history of) - Corneal epithelial defect - Corneal erosion - Recurrent corneal erosion |
|  | Corneal Dystrophies | - Anterior basement membrane dystrophy (ABMD) - Endothelial corneal dystrophy - Fuchs dystrophy - Lattice dystrophy - Map-dot-fingerprint - Corneal epithelial and basement membrane dystrophy |
|  | Corneal Grafts or Stem Cell Deficiency | - H/o cornea transplant - Cornea replaced by transplant - Corneal transplant status - History of deep anterior lamellar keratoplasty - History of Descemet membrane endothelial keratoplasty (DMEK) - Failed corneal transplant - Limbal stem cell deficiency - Mechanical complication due to cornea graft - Other complication of corneal transplant - Post-corneal transplant - Presence of corneal transplant - Rejection of corneal transplant (corneal graft rejection) - Corneal graft malfunction |
|  | Corneal Scar | - Corneal opacity - Scar with opacity - Subepithelial haze - Corneal neovascularization |
|  | Corneal Ulcer | - Central corneal ulcer - Corneal ulcer or ulceration (including herpetic, with hypopyon) - Marginal corneal ulcer - Marginal keratitis |
|  | Dry Eye, Blepharitis, or Meibomian Gland Dysfunction | - Chronically dry eye - Dry eye syndrome due to meibomian gland dysfunction - Dry eye syndrome - Dry eyes due to decreased tear production - Keratitis sicca (due to decreased tear production, not due to Sjogren’s syndrome) - Sjogren’s syndrome (with keratoconjunctivitis sicca - with other organ involvement) - Squamous blepharitis - Ulcerative blepharitis - Tear film insufficiency - Ocular rosacea |
|  | Exposure Keratopathy | - Exposure keratoconjunctivitis |
|  | Foreign Body Sensation and Photophobia | - Photophobia - Irritation of eyes - Sensation of foreign body in eye |
|  | Glare | - Glare sensitivity |
|  | Infectious Keratitis | - Acanthamoeba keratitis |
|  | Keratitis, Not Specified | - Interstitial Keratitis - Keratitis - Superficial punctate keratitis - Filamentary keratitis - Punctate epithelial keratopathy |
|  | Keratoconus and Ectasias | - Keratoconus (w/ & w/o acute hydrops) - Pellucid marginal degeneration - Corneal delle |
|  | Lens Dislocation | - Dislocated intraocular lens (initial encounter, subsequent encounter, sequela) - Dislocation of IOL into vitreous - Posterior dislocation of IOL - Mechanical complication due to intraocular lens implant - Uveitis-glaucoma-hyphema syndrome - Anterior dislocation of lens Ectopia lentis - Lens dislocation and subluxation subluxation of lens - Traumatic dislocation of lens |
|  | Neurotrophic Cornea | - Neurotrophic Keratitis - Neurotrophic cornea |
|  | Corneal Diseases, Not Otherwise Specified | - Bitot’s spots - Phlyctenular conjunctivitis - Chemosis of conjunctiva - Detachment of Descemet’s membrane - Conjunctival concretions - Conjunctival cyst - Conjunctivochalasis - Irregular contour of cornea - Keratic precipitates - Keratopathy - Superior limbic keratoconjunctivitis |
|  | Peripheral Corneal Diseases | - Peripheral ulcerative keratitis - Corneal melt - Corneal thinning |
|  | Posterior Capsular Opacification | - After-cataract - Posterior Capsule Opacification - Pupillary Membrane - Secondary cataract |
|  | Pseudophakia | - S/p Cataract Extraction and Insertion of Intraocular Lens |
|  | Pterygium and Pinguecula | - Pterygium - Pinguecula |
|  | Refractive Surgery, History of | - History of laser refractive surgery - S/p LASIK (laser assisted in situ keratomileusis) - S/p Photorefractive Keratectomy (PRK) |
|  | Viral Conjunctivitis | -Viral conjunctivitis |
|  | Viral Keratitis | - Herpetic keratitis - Herpes Zoster (keratitis, stromal keratitis, keratoconjunctivitis, pseudodendrites) - HSV keratitis (stromal, epithelial, dendritic) - herpes stromal keratitis |
| **Glaucoma** | Angle Closure Glaucoma | - Angle-closure glaucoma (mild, moderate, severe, indeterminate stage secondary) - Acute angle-closure glaucoma - Acute primary angle-closure glaucoma - Chronic angle-closure glaucoma - Chronic primary angle-closure glaucoma - Intermittent angle closure glaucoma - Residual stage of angle closure glaucoma |
|  | Angle Closure Glaucoma Suspect | - Anatomical narrow angle - Primary angle closure of both eyes without glaucoma damage - Primary angle closure suspect - Narrow angle glaucoma suspect |
|  | Aniridia | - glaucoma with aniridia |
|  | Congenital or Juvenile Glaucoma | - Congenital glaucoma - Glaucoma associated with anterior segment anomaly - Glaucoma of childhood - Juvenile OAG (open angle glaucoma) - Glaucoma due to congenital lens dislocation - Juvenile open angle glaucoma associated with mutation in MYOC gene |
|  | Glaucoma Suspect | - At high risk for open angle glaucoma - Ocular hypertenstion - Borderline glaucoma (with ocular hypertension) - Cupping of optic disc - Elevated IOP - Glaucoma suspect - OAG (open-angle glaucoma) suspect - Open angle glaucoma suspect with borderline findings - Open angle with cupping of optic discs - Optic cupping - Raised intraocular pressure - Glaucomatous cupping of optic disc |
|  | Hypotony or Phthisis Bulbi | - Phthisis bulbi - Hypotony (due to fistula) - Secondary hypotony |
|  | Inflammatory Glaucoma | - Uveitic glaucoma (mild, moderate, severe, indeterminate, unspecified stage) - Glaucoma secondary to eye inflammation |
|  | Neovascular Glaucoma | - Neovascular glaucoma (NVG) - Rubeosis iridis |
|  | Other Glaucomatous Disorders, Not Otherwise Specified | - Glaucoma secondary to drugs - Glaucoma due to silicone oil steroid-induced glaucoma - Glaucoma following surgery - Glaucoma associated with ocular trauma - Traumatic glaucoma - Low-tension glaucoma - Aphakic glaucoma - Angle recession - Glaucoma due to combination of mechanisms - End stage glaucoma - Other glaucoma - Malignant glaucoma |
|  | Phakomatoses | - Port-wine stain of face - Sturge-weber syndrome - Neurofibromatosis type I |
|  | Pigmentary Glaucoma and Suspects | - Pigmentary dispersion syndrome - Pigmentary glaucoma (mild, moderate, severe, indeterminate, unspecified stage) |
|  | Primary Open Angle Glaucoma | - Primary open angle glaucoma (mild, moderate, severe, indeterminate, unspecified stage) Chronic open angle glaucoma (mild, moderate stages) - Open angle glaucoma with borderline intraocular pressure - Open angle glaucoma (mild, moderate, severe, indeterminate, unspecified stages) - Open angle glaucoma cupping of optic discs |
|  | Pseudoexfoliation Glaucoma | - Capsular glaucoma with pseudoexfoliation (PXF) (mild, moderate, indeterminate stage) - Pseudoexfoliation (PXF) glaucoma (mild, moderate, indeterminate stage) - Pseudoexfoliation of lens capsule |
|  | Secondary Glaucomas | - Secondary angle closure glaucoma (severe, indeterminate stage) - Secondary glaucoma (mild, moderate, severe, indeterminate, unspecified stage) - Secondary open angle glaucoma, glaucoma associated with underlying disease - glaucoma secondary to other eye disorders - Secondary glaucoma due to combined mechanisms |
| **Low Vision** | Legal Blindness and Low Vision | - Blind painful eye - Legal blindness - Low vision - Blind painful eye with normal vision in contralateral eye |
| **General Ophthalmology - Trauma** | Closed globe injury | - Blunt eye trauma (initial, subsequent encounter, sequela) eye injury non-penetrating - Ocular trauma (initial, subsequent encounter) - Traumatic injury of globe |
|  | Collagen vascular disease screening | -Marfan syndrome  -Ehlers Danlos syndrome |
|  | Corneal trauma | - Corneal perforation - Corneal foreign body (initial encounter, with residual material) - Corneal laceration - Foreign body in conjunctival sac - Chemical burn of cornea (initial, subsequent encounter) - Chemical injury to eye - Conjunctival laceration - Traumatic conjunctivitis - Abrasion of conjunctiva - Chemical burn due to alkali conjunctiva or cornea - Thermal injury to conjunctiva or cornea |
|  | Endophthalmitis | - Infectious endophthalmitis - Fungal endophthalmitis |
|  | Eye pan | - Pain around eye - Pain in eye - Pain in periorbital region - Pain of orbit - Ocular pain - History of eye pain - Acute post-operative pain |
|  | Eyelid and canalicular trauma | - Eyelid laceration (initial, subsequent, sequela) - Canalicular laceration - Abrasion of eyelid - Facial laceration |
|  | Family History of Eye Disease Screening Exam | - Encounter for ocular screening examination |
|  | Hypertension | - Essential hypertension |
|  | Hyphema | - Traumatic hyphema |
|  | Intraocular Foreign Body | - Intraocular foreign body (magnetic, subsequent) |
|  | Iris Abnormalities | - Progressive iris atrophy - Iridocorneal endothelial syndrome - Iris bombe - Synechiae of iris - Posterior synechiae - Tear of iris stroma - Lisch nodules - Degeneration of ciliary body |
|  | Ocular Surgery History, Not Otherwise Specified | - Glaucoma shunt device - Post-procedural blebitis of eye - Bleb associated endophthalmitis - Cataract fragments in eye following cataract surgery - S/p evisceration - Anophthalmos (acquired) - History of eye enucleation - Broken suture - Eroded suture - Suture retraction - Post-operative state - Mechanical complication due to implant |
|  | General Ophthalmology Conditions, Not Otherwise Specified | -Patient left without being seen  -Pre-operative visit |
|  | Systemic Medical Conditions | -Anemia  -Obesity  -Sleep apnea  -Homelessness  -Pregnancy  -Hypothyroidism  -Dizziness  -Angioedema |
|  | Pupillary Abnormalities | - Horner syndrome - Relative afferent pupillary defect - Anisocoria - Tonic pupil - Traumatic mydriasis |
|  | Red Eyes | - Ocular Hyperemia of both eyes |
|  | Refractive error | - Anisometropia - Astigmatism following corneal transplant - Astigmatism (with presbyopia - Irregular, regular, myopic, hyperopic) - high myopia - Hyperopia (with astigmatism, presbyopia) - Myopia (with astigmatism, presbyopia) - Pathologic myopia - Presbyopia - Refractive error - High myopia - Impaired visual accommodation |
|  | Research Patient Visit | - Enrolled in clinical trial of drug - Examination of participant in clinical trial - Research exam - Research study participant - Research subject |
|  | Ruptured Globes | - Rupture of globe (following blunt trauma, with uveal prolapse   initial, subsequent, sequela) |
|  | Screening Exam for an Eye Condition | - Encounter for medical screening examination |
|  | Subconjunctival Hemorrhage | - Subconjunctival hematoma |
|  | Systemic Malignancy | - Mesothelioma - Malignant neoplasm of breast - Lung nodule - Prostate cancer metastatic to central nervous system - Lymphoma of lymph nodes in pelvis - Chronic myeloid leukemia - Metastasis to eye - Secondary malignant melanoma of lung |
|  | Systemic Neuropsychiatric Disorders | - Agorophobia with panic attacks - Anxiety - Claustrophobia - Post-concussion syndrome - Inflammatory neuropathy - Paraneoplastic neurologic disorder - Anoxic brain injury, seizures |
|  | Vasculitides, Ocular Exam to Rule Out Complications | -Granulomatosis with polyangiitis  -Temporal arteritis  -Takayasu arteritis  -Churg Strauss  -Lupus  -Systemic lupus erythematosis |
|  | Visual Disturbance or Distortion | - Blurred vision - blurry vision - decreased vision - vision changes - Vision loss - Visual disturbance - Visual impairment - Visual symptoms - Subjective visual disturbance - Transient monocular blindness - Transient vision disturbance - Transient visual disturbance - Binocular visual disturbance - Asthenopia - Eye strain - Loss of vision - Visual distortions of shape and size - Amaurosis fugax - Moderate vision impairment - Impaired contrast sensitivity |
| **Neuro-ophthalmology** | Color Vision Disorder | -Achromatopsia  -Red green color deficiency |
|  | Facial Nerve Palsy | - Facial nerve palsy - Bell’s palsy |
|  | Facial Spasm | - Meige syndrome - Blepharospasm (benign essential) - Hemifacial spasm (clonic) - ocular torticollis |
|  | Third Nerve Palsy | - Total 3^rd^ nerve palsy |
|  | Fourth Nerve Palsy | - Trochlear nerve palsy |
|  | Migraine | - Atypical Migraine - Migraine (with or without aura & with or without status miraninosus) - Intractable migraine - Ophthalmic migraine - Vestibular migraine - Ocular migraine - Acute post-traumatic headache - Hemicrania continua - New daily persistent headache - Temporal headache |
|  | Multiple Sclerosis | - MS (multiple-sclerosis) |
|  | Myasthenia Gravis | - Myasthenia gravis - Ocular myasthenia - Generalized myasthenia gravis |
|  | Neuralgia | - Supraorbital neuralgia - Trigeminal neuralgia - Occipital neuralgia - Post-herpetic neuralgia |
|  | Optic Neuritis | - Optic papillitis - Retrobulbar optic neuritis - Optic neuritis with optic disc edema |
|  | Optic Neuropathies | - Optic neuropathy - Hereditary optic neuropathy - Ischemic optic neuropathy (neuritis) - Non-arteritic anterior ischemic optic neuropathy - Arteritic anterior ischemic optic neuropathy |
|  | Neuro-ophthalmic Tumors | - Astrocytoma - Pilocytic astrocytoma - Meningioma - Pituitary macroadenoma - Pituitary adenoma - Craniopharyngioma - Hemangiopericytoma ganglioglioma - Brain tumor - Pituitary tumor - Rathke’s cyst - Pituitary cyst - Brain mass |
|  | Cerebrovascular Diseases | - Venous sinus thrombosis (cerebral, dural) - Transient ischemic attack - Cerebrovascular accident due to embolism - Occipital stroke - Carotid artery disorder - Chiari malformation (Type I & II) |
|  | Nystagmus | - Spasmus nutans - Nystagmus |
|  | Optic Atrophy | - Optic disc pallor - Temporal pallor of optic disc |
|  | Optic Disc Edema | - Papilledema (due to increased intracranial pressure) - Optic nerve swelling - Optic nerve edema |
|  | Other Neuro-ophthalmic Disorders, Not Otherwise Specified | - Vestibular dysfunction - Parinaud’s syndrome - Double elevator palsy - Nyctalopia - Superior oblique tendon sheath syndrome - Limited ocular motility |
|  | Optic Nerve Disorders, Not Otherwise Specified | - Pseudopapilledema - Other disorders of optic disc - Neural-optical lesion - Optic nerve glioma - Anomalous optic nerve - Chorioretinal coloboma - Optic nerve hypoplasia - Myelinated optic nerve fiber layer - Primary meningioma of optic nerve sheath |
|  | Intracranial Hypertension | - Pseudotumor cerebri - Idiopathic intracranial hypertension - Obstructive hydrocephalus - S/p VP shunt |
|  | Scotomas and Visual Field Abnormalities | - Visual field defect - Visual field loss - Paracentral scotoma - Central scotoma - Absolute scotoma - Arcuate scotoma - Altitudinal scotoma - Enlarged blind spot - Visual field constriction (concentric) - Loss of peripheral visual field - Other localized visual field defect - Homonymous hemianopia, bitemporal hemianopsia - Tunnel visual field constriction - Scintillating scotoma - Visual loss, one eye no light perception |
|  | Sixth Nerve Palsy | - Abducens (sixth) nerve palsy - Abducens nerve paresis - Traumatic injury of abducens nerve |
| **Oncology** | Choroidal Mass | - Choroidal melanoma - Choroidal malignant melanoma - Malignant melanoma of choroid - Hemangioma of choroid - Neoplasm of uncertain behavior of choroid |
|  | Iris and Ciliary Body Melanoma | - Ciliary body tumor - malignant melanoma of ciliary body |
|  | Choroidal Nevus | - Choroidal nevus - Nevus of choroid |
|  | Primary Acquired Melanosis | - Melanosis of conjunctiva - PAM (primary acquired melanosis) |
|  | Retinoblastoma | -Retinoblastoma |
| **Oculoplastic Surgery** | Benign Eyelid Lesions | - Benign tumor of eyelid including canthus - Conjunctival papilloma - Sebaceous cyst of eyelid - Nevus of eyelid - Skin tag - Lesion of canthus - Eyelid lesion (upper lid / lower lid) |
|  | Chalazion | - Chalazion (upper / lower eyelid & unspecified) |
|  | Dermatochalasis | - Dermatochalasis |
|  | Eyelid Malposition | - Entropion of eyelid - Cicatricial entropion - Ectropion of eyelid - Senile ectropion - Paralytic ectropion - Cicatricial lagophthalmos - Paralytic lagophthalmos |
|  | Eyelid Malignancy | - Basal Cell Carcinoma - Squamous cell cancer |
|  | Hordeolum | - Hordeolum externum - Hordeolum internum - Meibomianitis |
|  | Orbital Inflammation or Infection | - Mucormycosis - Abscess of right orbit - Orbital cellulitis - Orbital inflammation inflammatory orbital pseudotumor - Cellulitis - Preseptal cellulitis |
|  | Oculoplastic Disorders, Not Otherwise Specified | - Floppy eyelid syndrome - Edema of eyelid - Swelling of eyelid - Eyelid abnormality - Ecchymosis of eye - Enophthalmos due to silent sinus syndrome - Periorbital swelling - Nasopharyngeal mass - Apraxia of eyelid opening |
|  | Nasolacrimal Duct Obstruction | - Epiphora (due to excess lacrimation) - Dacryocystitis (acute) - Canaliculitis (acute) - Nasolacrimal duct obstruction (neonatal, congenital, acquired) - Punctal stenosis - Presence of punctal plug |
|  | Orbital Fractures | - Closed fracture of frontal bone - Orbital floor (blow-out) fracture - Open orbital fracture - Closed fracture of orbit with routine healing |
|  | Ptosis | - Ptosis of eyelid - Involutional ptosis (acquired) - Mechanical ptosis - Myogenic ptosis - Congenital ptosis |
|  | Thyroid Eye Disease | - Proptosis (due to thyroid disorder) - Graves ophthalmopathy - Graves disease - Thyroid eye disorder - Restriction of extraocular movement due to thyroid disorder |
|  | Trichiasis | - Trichiasis of eyelid without entropion - Trichiasis (upper, lower, unspecified) |
|  | Orbital Tumors | - Lymphangioma - Orbital mass - Sarcoma of orbit |
| **Retina** | Central Serous Chorioretinopathy | - Central serous chorioretinopathy - Central Serous retinopathy (CSR) - Central serous retinopathy with small retinal pigment epithelial detachment |
|  | Choroidal Neovascularization | - Choroidal neovascularization (due to chorioretinitis, due to angioid streaks, due to pathologic myopia) - Choroidal neovascular membrane / CNVM - Classic choroidal neovascular membrane - Neovascular membrane of choroid artery - Retinal neovascularization - Idiopathic choroidal neovascularization |
|  | Choroidal Scar | - Chorioretinal scar(s) - Retinal scar - Peripheral chorioretinal scars - Chorioretinal scar after retinal detachment surgery |
|  | Cystoid Macular Edema | - Cystoid macular degeneration (of retina) - Cystoid macular edema (CME) - Macular edema - Retinal edema - Uveitis related cystoid macular edema - Postoperative cystoid macular edema - CME following cataract surgery - Irvine-gass syndrome |
|  | Diabetic Eye Exam | - Uncontrolled (type 1 or type 2) diabetes - Controlled (type 1 or type 2 diabetes (with or without) complication - Uncontrolled type 1 or type 2 diabetes (with or without) complication - Type 1 or Type 2 diabetes mellitus (with or without) complication, (with or without) long-term current use of insulin - (Type 1 or Type 2) diabetes mellitus (with or without) complications (or with unspecified complications) (with or without) long-term current use of insulin - Prediabetes - Diabetes (type 1 or type 2), no ocular involvement - Diabetic eye exam - Diabetes mellitus (type 1 or type 2), insulin-dependent - Diabetes mellitus without complication - Diabetes mellitus (type 1 or type 2) without retinopathy - Type 1 or Type 2 diabetes mellitus without ophthalmic manifestations |
|  | Epiretinal Membrane | - Macular pucker - Epiretinal membrane |
|  | Exudative Age Related Macular Degeneration, Active | - Wet AMD with active choroidal neovascularization - Exudative AMD with active choroidal neovascularization |
|  | Exudative Age Related Macular Degeneration, Inactive | - Exudative AMD with inactive choroidal neovascularization - Exudative AMD with inactive scar - Wet AMD with inactive scar - Exudative AMD unspecified stage |
|  | Hypertension Retinopathy | - Hypertensive retinopathy (grades 1-4) |
|  | Macular Holes | - Full thickness macular hole - Macular hole - Early stage macular hole - Lamellar macular hole |
|  | Macular Scars and Telangectasias | - Retinal telangiectasia - Type 2 macular telangiectasia - Idiopathic macular telangiectasia type 1 - Macular scar - Macular scars of posterior pole (post-inflammatory / post-traumatic) |
|  | Myopia- Associated Retinal Disorders | - Uncomplicated degenerative myopia - Degenerative myopia with choroidal neovascularization - Degenerative myopia with retinal detachment - Degenerative myopia with foveoschisis - Severe myopia with complication - Myopic macular degeneration |
|  | Non-Exudative Age-Related Macular Degeneration | - Age-Related macular degeneration - Intermediate stage dry AMD - Non-exudative AMD, intermediate dry stage - Advanced dry AMD (with or without) subfoveal involvement - (Early, intermediate, or advanced) stage dry AMD - Intermediate AMD - Intermediate stage nonexudative AMD - Non-exudative AMD, advanced atrophic (with or without) subfoveal involvement - Drusen (degenerative) of retina or macula - Retinal or macular drusen - AMD with central geographic atrophy |
|  | Non-Proliferative Diabetic Retinopathy with Cystoid Macular Edema | - (Mild, moderate, severe) NPDR with macular edema associated with (Type 1 or Type 2) diabetes mellitus (with or without) long-term current use of insulin - Diabetic macular edema - Diabetic retinopathy with macular edema associated with diabetes mellitus due to underlying condition (mild, moderate, severe, unspecified severity) - Diabetic visual loss (moderate vision impairment, total vision impairment, blindness of both eyes) with macular edema associated with (Type 1 or Type 2 diabetes) - Controlled (type 1 or type 2) diabetes mellitus affected by (mild, moderate, severe) NPDR and macular edema - Uncontrolled (type 1 or type 2) diabetes mellitus affected by moderate NPDR and macular edema |
|  | Non Proliferative Diabetic Retinopathy without Cystoid Macular Edema | - (Mild, moderate, severe) NPDR (without macular edema or macular edema presence unspecified) associated with (Type 1 or Type 2 diabetes) (with or without) long-term current use of insulin - Diabetes mellitus with background retinopathy - (Controlled or uncontrolled) NPDR (Type 1 or Type 2) diabetes with severe NPDR macular edema presence unspecified |
|  | Other Vitreoretinal Disorders, Not Otherwise Specified | - Macular areolar choroidal atrophy - Chorioretinal degeneration - Serpiginous choroidal systrophy - Suprachoroidal hemorrhage - Choroidal rupture - Choroidal detachment - Choroidal lesion - Choroidal folds - Choroidal granuloma - Macular (subretinal) hemorrhage - Retinal hemorrhage (noted on examination) - Subretinal hemorrhage - Idiopathic polypoidal choroidal vasculopathy - Retinal pigment epitheliopathy - Macular pigment epithelial tear - (Retinal or Macular) pigment epithelial detachment - (Macular) RPE mottling - Congenital hypertrophy of Retinal pigment epithelium (CHRPE) - Proliferative retinopathy / vitreoretinopathy (non-diabetic) - Commotio retinae (initial, subsequent encounter) - History of vitrectomy - Family history of retinal detachment - Other specified retinal disorders - Maculopathy - Multiple defects of retina without detachment - Purtscher’s retinopathy - Retinopathy - Retinal microaneurysm - Coat’s disease - Angioid streaks - Retinal degeneration - Retinal lesion - Retinal macular atrophy - Stickler’s syndrome - Radiation retinopathy - Diabetic visual loss - Autoimmune retinopathy - Bilateral diffuse uveal melanocytic proliferation - Retinal hemangioblastomatosis - Radiation damage to optic nerve - Post-radiation retinopathy |
|  | Peripheral Retinal Degenerations | - Lattice degeneration (of retina) - Microcystoid degeneration of retina - Cobblestone retinal degeneration - Pavingstone retinal degeneration - Senile reticular pigmentary degeneration - Cystic retinal tuft - Peripheral retinal degeneration |
|  | Photopsias | - Flashing lights seen - vitreous flashes |
|  | Posterior Vitreous Detachments, Floaters, or Syneresis | - Floaters in visual field - Vitreous floaters - Vitreous opacities - Vitreous syneresis - Floaters - Symptomatic Posterior vitreous detachment (PVD) - Vitreous degeneration (and/or detachment) - Posterior vitreous detachment (PVD) - Vitreoretinal degeneration - Degeneration of posterior vitreous body |
|  | Proliferative Diabetes with Cystoid Macular Edema | - Proliferative diabetic retinopathy with macular edema associated with (Type 1 or Type 2) diabetes mellitus (with or without or unspecified) long-term current use of insulin - Proliferative diabetic retinopathy associated with diabetes mellitus due to underlying condition (macular edema presence unspecified, with traction retinal detachment involving macula) - (controlled or uncontrolled) (Type 1 or Type 2) diabetes mellitus with macular edema (with or without) long-term current use of insulin - PDR with macular edema determined by examination associated with (type 1 or type 2 diabetes mellitus) |
|  | Proliferative Diabetes without Cystoid Macular Edema | - Proliferative diabetic retinopathy without macular edema (or macula edema presence unspecified) associated with (Type 1 or Type 2) diabetes mellitus (with or without) long-term current use of insulin - stable proliferative diabetic retinopathy associated with (type 1 or type 2) diabetes, (with or without) long-term current use of insulin - Proliferative diabetic retinopathy associated with diabetes mellitus due to underlying condition (macular edema presence unspecified, with traction retinal detachment involving macula) - Proliferative diabetic retinopathy with combined traction and rhegmatogenous retinal detachment (involving macula, not involving macula), associated with (type 1 or type 2) diabetes mellitus - Proliferative diabetic retinopathy with traction retinal detachment (involving macula, not involving macula), associated with (type 1 or type 2) diabetes mellitus |
|  | Rhegmatogenous Retinal Detachments | - (Macula-off or Macula-on) rhegmatogenous retinal detachment - Retinal detachment with (retinal break, single break, multiple breaks, multiple retinal tears) - Rhegmatogenous retinal detachment - Retinal detachment with giant retinal tear - Recent subtotal retinal detachment - Total retinal detachment - Retinal detachment, old (total or subtotal) - Retinal detachment |
|  | Retinal Artery Occlusions | - Central retinal artery occlusion, - Partial retinal artery (branch) occlusion - Branch macular artery occlusion - Branch retinal artery occlusion |
|  | Retinal Dystrophies | - Macular dystrophy - Pigmentary retinopathy - Retinitis pigmentosa - Stargardt’s disease - Dystrophies primarily involving the retinal pigment epithelium - Adult onset vitelliform macular dystrophy - Macular pattern dystrophy - Pattern dystrophy of macula |
|  | Retinal Tears or Holes, Non-Macular | - Retinal break - Horseshoe retinal tear - Retinal tear - Peripheral retinal hole - Retinal hole |
|  | Retinal Vein Occlusions with Macular Edema | - (Central or Branch) retinal vein occlusion with macular edema - Hemispheric retinal vein occlusion with macular edema - Central retinal vein occlusion with neovascularization |
|  | Retinal Vein Occlusions without Macular Edema | - Stable central retinal vein occlusion - Hemispheric retinal vein occlusion - (Central or Branch) retinal vein occlusion with neovascularization - Retinal venous engorgement |
|  | Ocular Exam in Patients with Hemoglobinopathies | -Hemoglobin SC disease  -Sickle Cell  -Thalassemia |
|  | Vitreomacular Traction Syndrome | - Vitreomacular adhesion - Vitreomacular traction syndrome - Vitreomacular traction |
|  | Vitreous Hemorrhage | - Vitreous hemorrhage due to (type 1 or type 2 diabetes mellitus) - Vitreous hemorrhage |
| **Pediatric Ophthalmology** | Albinism | -Albinism  -Tyrosinase deficiency  -Oculocutaneous albinism |
|  | Amblyopia | - Amblyopia - Deprivation amblyopia - Refractive amblyopia - Anisometropic amblyopia |
|  | Congenital Anatomic Abnormalities | - Peters plus syndrome - Anophthalmia - Anterior segment dysgenesis |
|  | Disorders of Convergence, Divergence, or Accomodation | - Convergence insufficiency - Divergence insufficiency - Accomodative insufficiency |
|  | Diplopia | - Diplopia - Binocular vision disorder with diplopia - Double vision - Monocular diplopia |
|  | Duane syndrome | -Duane syndrome (types 1-3) |
|  | Esotropia | - Esotropia - Pseudoesotropia - alternating esotropia - intermittent esotropia - Accommodative esotropia |
|  | Exotropia | - Intermittent exotropia - Alternating Exotropia - Sensory deprivation exotropia - Exotropia |
|  | Phorias | - Exophoria - Esophoria |
|  | Pediatric Ophthalmic Disorders, Not Otherwise Specified | - Abnormal red reflex - Restrictive strabismus - Spasm (infantile) - Leukocoria |
|  | Retinopathy of Prematurity | - Retinopathy of prematurity (Stage 1 – 5, Zone 1-3, with or without plus disease) |
|  | Ocular Exam in Patients with Chromosomal Abnormalities or Inborn Errors of Metabolism | -Down syndrome  -Turner Syndrome  -Biotinidase deficiency  -Cystic fibrosis |
|  | Vertical Strabismus | - Vertical diplopia - Hypertropia |
| **Uveitis** | Birdshot Chorioretinitis | - Birdshot choroidopathy - Birdshot chorioretinitis |
|  | Cicatrizing Disorders of the Conjunctiva | - Cicatricial lagophthalmos - Conjunctival scarring - Benign mucous membrane pemphigoid with ocular involvement - Mucous membrane pemphigoid - Ocular cicatricial pemphigoid |
|  | Cytomegalovirus Retinitis | - CMV retinitis |
|  | High Risk Medication Use | - Encounter for monitoring NSAID therapy - NSAID long term use - Long term (current) use of systemic steroids - On prednisone therapy - On methotrexate therapy - Long term use of hydroxychloroquine - Encounter for eye exam due to high risk medication - Long term current use of immunosuppressive drug - Long term use of high risk medication - Encounter for long term (current) use of medications - Long-term use of Plaquenil - on cellcept therapy - Long term use of immunosuppressant medication - high risk medication use |
|  | Episcleritis | -Episcleritis |
|  | Herpetic Disease, Non-corneal | - Herpes infection - Herpes simplex virus infection - Ramsay-hunt syndrome - Herpes zoster (with & without complication) - Herpes zoster ophthalmicus - Iridocyclitis due to herpes zoster - Zoster scleritis - Shingles of eyelid - Acute retinal necrosis |
|  | Ocular Exam in Patients with HIV | - HIV disease - HIV infection - AIDS (Acquired Immunodeficiency Syndrome) |
|  | HLA B27+ Associated Uveitis | - Iridocyclitis associated with HLA-B27 positivity |
|  | Juvenile Idiopathic Arthritis-Associated Uveitis | - Juvenile Rheumatoid Arthritis (JRA) - Chronic polyarticular juvenile rheumatoid arthritis - Juvenile idiopathic arthritis - Juvenile idiopathic arthritis associated chronic anterior uveitis |
|  | Multifocal Choroiditis with Panuveitis | - Multifocal choroiditis - Multifocal choroiditis and panuveitis |
|  | Uveitis, Not Otherwise Specified | - Granulomatous uveitis - Uveitis - Chronic uveitis - History of uveitis |
|  | Posterior Uveitis | - Posterior uveitis - Choroiditis - Chorioretinitis - Serpiginous choroiditis - Retinal vasculitis - Acute zonal occult outer retinopathy (AZOOR) - Focal chorioretinal inflammation of posterior pole - Disseminated choroiditis and chorioretinitis - Generalized disseminated chorioretinal inflammation |
|  | Presumed Ocular Histoplasmosis Syndrome | -Presumed ocular histoplasmosis syndrome |
|  | Primary Intraocular Lymphoma | - Ocular lymphoma - Vitreoretinal lymphoma |
|  | Punctate Inner Choroidopathy | -Punctate Inner Choroidopathy |
|  | Scleritis | - Scleritis - Scleritis and Episcleritis - Scleritis due to Granulomatosis with polyangiitis - anterior scleritis - posterior scleritis - necrotizing scleritis - scleromalacia - sclerokeratitis - sclerouveitis |
|  | Syphilitic Uveitis | - Positive Serology for Syphilis - Syphilitic uveitis |
|  | Toxoplasmosis Retinitis | - Toxoplasma chorioretinitis |
|  | Undifferentiated Anterior Uveitis | - Iridocyclitis (acute or subacute) - Acute anterior uveitis - Acute iridocyclitis - Acute iritis - Anterior uveitis - Recurrent iritis - Recurrent (acute) iridocyclitis - Chronic iridocyclitis - Chronic anterior uveitis - Chronic iritis - Chronic uveitis - Iridocyclitis, iritis |
|  | Undifferentiated Intermediate Uveitis | - Posterior cyclitis - Pars planitis - Posterior cyclitis - Intermediate uveitis |
|  | Undifferentiated Panuveitis |  |
|  | Uveitis- Associated with Other Rheumatological Disorders | - Reactive arthritis - Mixed connective tissue disease - Behcet’s disease - Ankylosing spondylitis (of cervical, lumbar region) - Rheumatoid arthritis of multiple sites with negative Rheumatoid factor - ANA positive - Rheumatoid factor positive - HLA-B27 positive - Sarcoidosis - Neurosarcoidosis |
|  | Vogt-Koyanagi-Harada Syndrome and Sympathetic Ophthalmia | - Sympathetic ophthalmitis - Vogt-Koyanagi-Harada syndrome - Vogt-Koyanagi syndrome |
